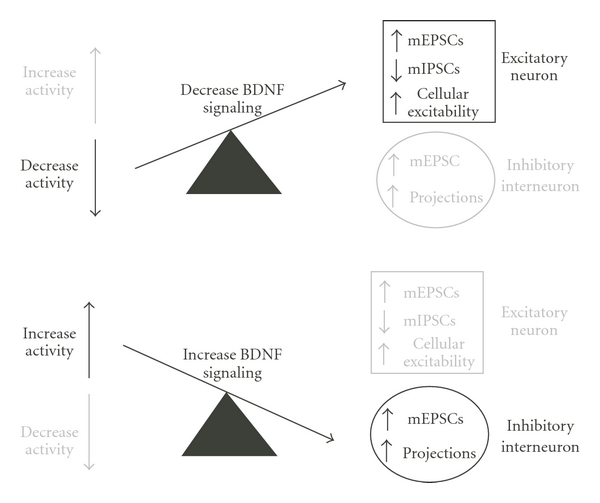# Mechanisms of GABAergic Homeostatic Plasticity

**DOI:** 10.1155/2011/489470

**Published:** 2011-08-17

**Authors:** Peter Wenner

**Affiliations:** Department of Physiology, Emory University, School of Medicine, 615 Michael Street, Room 601, Atlanta, GA 30322, USA

## Abstract

Homeostatic plasticity ensures that appropriate levels of activity are maintained through compensatory adjustments in synaptic strength and cellular excitability. For instance, excitatory glutamatergic synapses are strengthened following activity blockade and weakened following increases in spiking activity. This form of plasticity has been described in a wide array of networks at several different stages of development, but most work and reviews have focussed on the excitatory inputs of excitatory neurons. Here we review homeostatic plasticity of GABAergic neurons and their synaptic connections. We propose a simplistic model for homeostatic plasticity of GABAergic components of the circuitry (GABAergic synapses onto excitatory neurons, excitatory connections onto GABAergic neurons, cellular excitability of GABAergic neurons): following chronic activity blockade there is a weakening of GABAergic inhibition, and following chronic increases in network activity there is a strengthening of GABAergic inhibition. Previous work on GABAergic homeostatic plasticity supports certain aspects of the model, but it is clear that the model cannot fully account for some results which do not appear to fit any simplistic rule. We consider potential reasons for these discrepancies.

## 1. Introduction

Alterations in the influence of inhibitory GABAergic circuits can have a profound impact on the excitability of neural network function, and have been associated with hyperexcitable conditions such as epilepsy [1, 2]. Recent work has identified what may be one of the most important processes in ensuring that networks maintain appropriate activity levels; homeostatic plasticity is thought to maintain network spiking activity levels within a physiologically relevant range through compensatory adjustments in intrinsic cellular excitability, as well as excitatory and inhibitory synaptic strength [3–8]. These changes are induced following perturbations in spiking activity levels for many hours. This phenomenon has been identified in several systems, at different developmental stages, *in vitro* and to a lesser extent *in vivo*. When activity levels of cultured neuronal networks (cortical, hippocampal, spinal) are altered for days, cellular excitability and synaptic strength within the network are adjusted in a direction that appears to oppose the alteration in activity [9–14]. For instance, when spiking activity is blocked for 2 days, AMPAergic synaptic strength increases and GABAergic synaptic strength decreases in excitatory neurons. When network spiking activity is increased, AMPAergic synaptic strength decreases. In each case the amplitude of miniature postsynaptic currents (mPSCs) changed in a direction to compensate for the perturbation. 

**Figure 1:**
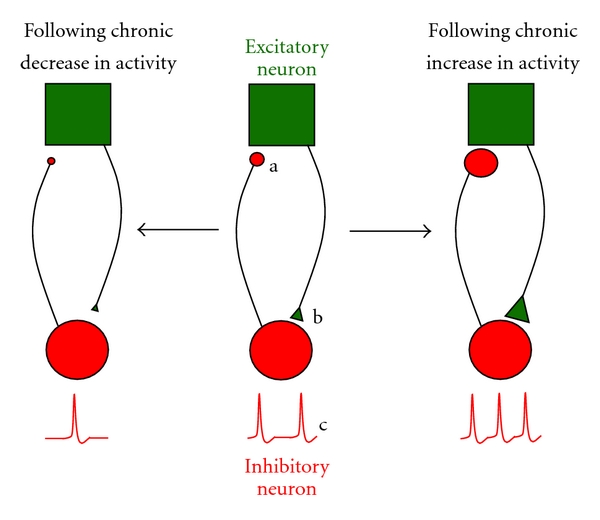


Several reviews have examined homeostatic plasticity, typically focusing on excitatory components within networks [3–6]. In this paper we will instead concentrate on the findings of homeostatic plasticity within GABAergic neurons and at GABAergic synapses onto excitatory neurons. Based on previous work studying homeostatic plasticity in the glutamatergic system, we make the simplistic prediction that inhibition would be reduced following network activity blockade and increased following increases in network activity. Therefore, following chronic reductions in network activity (Figure 1 left), we would expect compensatory weakening of both GABAergic synapses on excitatory neurons and glutamatergic synapses on inhibitory neurons and to see reductions in the intrinsic cellular excitability of inhibitory neurons. If network activity is increased for days (Figure 1 right), we would expect compensatory strengthening of both GABAergic synapses on excitatory neurons and glutamatergic synapses on inhibitory neurons and to observe increases in the intrinsic cellular excitability of inhibitory neurons. The review focuses on compensatory changes in cellular excitability and mPSC amplitude. 

## 2. Homeostatic Synaptic Plasticity of GABAergic Inputs to Excitatory Neurons

The most studied aspect of inhibition in homeostatic plasticity has examined the inhibitory GABAergic inputs to excitatory neurons (Figure 1(a)). Immunocytochemical studies gave the first indication that GABAergic circuits experienced homeostatic plasticity, as reduced visual input led to decreased cortical expression of GABA_A_ receptors, GABA, and GAD [15, 16]. Compensatory changes in the amplitude of GABAergic mPSCs have now been demonstrated in excitatory neurons following network activity perturbations in several different studies [10, 17–20]. These changes in GABAergic mPSC amplitude are often mediated by changes in the number of synaptic GABA_A_ receptors, and this is typically shown by quantitative immunocytochemistry [10, 20, 21]. In addition, compensatory changes in the vesicular inhibitory amino acid transporter, VIAAT, have been observed suggesting there are coordinated presynaptic contributions to homeostatic changes in mIPSC amplitude [18, 20, 21]. While these studies have shown that mIPSC amplitude is reduced following chronic activity blockade or increased following increased network activity, two studies suggest the opposite can occur. One study demonstrates that a subset of GABAergic inputs to hippocampal pyramidal cells are strengthened following activity block; however, the overall population of mIPSCs homeostatically scale downward [19]. Another study shows that *in vivo* application of TTX for 2 days resulted in an increase in mIPSC amplitude in pyramidal cells recorded from cortical slices [22], which also does not fit the simple homeostatic model. These studies highlight the need to carry out more homeostatic studies *in vivo*, as perturbations in living networks are likely to be more complicated in terms of network homeostasis, but crucial in elucidating the goals of homeostatic plasticity.

In a separate study where spiking activity was blocked *in vivo* for 2 days in the embryonic spinal cord, the result appeared to obey our homeostatic model. Following activity blockade, GABAergic mPSCs in excitatory motoneurons increased in amplitude [17]. These changes in GABAergic currents were compensatory because GABA was depolarizing and excitatory at this developmental stage. This is due to chloride accumulation through transporters expressed at these stages [23]. Interestingly, homeostatic increases in GABAergic mPSCs occurred through increased chloride accumulation, thus depolarizing the GABAergic reversal potential (E_GABA_) and enhancing the driving force for these currents [24]. Similarly, another study indirectly demonstrated that homeostatic changes in GABAergic currents could be produced by a shift in E_GABA_ [25]. In this study, activity was perturbed in hippocampal organotypic cultures and compensatory changes in GABAergic currents were observed in pyramidal cells at a stage when GABA was no longer excitatory. These findings are important for understanding the maturation of GABAergic synaptic strength but also may have implications for neuronal injury in mature circuits where the same depolarizing shifts in chloride reversal potential are observed following spinal cord injury, peripheral nerve injury, and traumatic brain injury [26–38]. It is tempting to speculate that following injury, homeostatic mechanisms may be engaged that produce the maladaptive increases in excitability associated with neuronal injury [30]. Consistent with this idea, work in a model of febrile seizure suggests the possibility that compensatory increases in GABAergic strength appear to promote hyperexcitability by triggering the hyperpolarization-activated current, I_h_ [39, 40]. 

Other studies in cultured networks demonstrated that homeostatic changes in mIPSC amplitude were not due to changes in E_GABA_ [10, 41]. However, these studies used whole-cell recordings to measure E_GABA, _which may dialyze intracellular Cl^−^ and mask the experimenter's ability to observe changes in GABAergic driving force. Future studies assessing homeostatic changes in mIPSCs could use perforated patch recordings or chloride indicators to resolve this issue.

Although not as common as homeostatic changes in mIPSC amplitude, homeostatic changes in mIPSC frequency have been reported. Increases or decreases in network activity have been shown to increase and decrease mIPSC frequency, respectively, in excitatory neurons [10, 20]. This appears to be mediated by changes in the number of GABAergic inputs to excitatory pyramidal cells. To a large extent, GABAergic mPSC amplitude and frequency in excitatory neurons follow the homeostatic model, strengthening after chronic increases in activity and weakening after activity blockade.

## 3. Homeostatic Plasticity in GABAergic Interneurons

Our homeostatic model predicts that AMPAergic synaptic inputs to GABAergic neurons will strengthen following increases in activity and weaken following activity blockade (Figure 1(b)). Using hippocampal cultures, it was shown that parvalbumin-expressing inhibitory interneurons (PV INs) increased mEPSC amplitude following chronic enhancement of activity levels and reduced mEPSC amplitude following activity blockade [42]. The changes in mEPSC amplitude were mediated by changes in the number of an AMPA receptor subunit, GLUA4, which was regulated homeostatically by neuronal activity-regulated pentraxin (NARP). Similarly, chronic increases in activity induced a strengthening of excitatory inputs to inhibitory interneurons in neocortical cultures, expressed presynaptically as an increase in the vesicular glutamate transporter, VGLUT2 [43]. Consistent with these findings, another study demonstrated that increasing BDNF levels, as occurs with increased network activity, led to an increase in mEPSC amplitude in inhibitory bipolar interneurons [41]. In this study, the increase in mEPSC amplitude was mediated by an increase in the sensitivity of the postsynaptic cell to glutamate, consistent with an increase in synaptic glutamate receptors. However, when spiking activity was blocked for days in several different cultured cortical networks, mEPSC amplitude was unaltered in multiple classes of inhibitory interneuron [8, 41, 44]. Thus far, the results are consistent with the idea that increased network activity levels triggered homeostatic increases in mEPSC amplitude in interneurons, but that mEPSC amplitude was typically unaltered by reductions in network activity. In none of these studies were changes in mEPSC frequency observed. Finally, we know of no homeostatic studies examining mIPSCs in inhibitory interneurons following activity perturbations.

Changes in interneuronal intrinsic cellular excitability (Figure 1(c)) following activity block have been described in 2 different cortical cultures. In both studies, intrinsic cellular excitability was increased following activity blockade in 3 different classes of inhibitory interneuron [44, 45]. One of the studies suggested that the increased excitability was the result of an increase in input resistance [44]. From a simplistic network perspective, increasing the excitability of an inhibitory neuron in an activity-blocked network is not what our homeostatic model would predict (Figure 1(c) left). The enhanced inhibition may be offset by the observation that pyramidal cells also have increased intrinsic excitability following activity blockade, but the finding underlines the complexity of the homeostatic process [22, 25, 44, 45]. It is possible that activity perturbations result in changes in synaptic strength that are homeostatic for the network, while changes in intrinsic cellular excitability are homeostatic from the perspective of the individual cell.

## 4. Evoked Responses between Inhibitory and Excitatory Neurons

We have focused on mPSCs because they provide a nice measure of a standard unit of synaptic strength. However, another potentially useful measure of synaptic strength is provided by looking at the functional connections between 2 components of the circuitry. The strength of the connections between inhibitory and excitatory neurons can be assessed through paired recordings, stimulating an inhibitory or excitatory neuron and recording a response in the other. The results of these studies have been somewhat mixed. When retinal activity is reduced *in vivo* by TTX infusion or lid suture, input to pyramidal cells in the visual cortex from inhibitory interneurons was homeostatically reduced in certain cases [46, 47]. In other cases reductions of visual input to cortical neurons resulted in a strengthening of both inhibitory inputs to pyramidal cells and pyramidal input to inhibitory neurons [46, 48]; from a network perspective, these results are opposite to that predicted by our model of homeostatic plasticity. One complication in these studies is that when visual input is perturbed *in vivo*, it is not always clear how this affects the activity of the visual cortical circuitry that is being studied; for instance, different results have been described when retinal activity is altered by lid suture versus TTX infusion [47]. However, in one study in neocortical organotypic cultures, where network activity was clearly blocked, changes in the strength of connections between excitatory and inhibitory neurons were not simplistically homeostatic [44].

## 5. BDNF

Brain-derived neurotrophic factor (BDNF) has been implicated in the signaling pathway for homeostatic plasticity of both glutamatergic and GABAergic systems. BDNF exerts its influence through changes in intrinsic cellular excitability, mEPSC and mIPSC amplitude and frequency. From these studies a pattern is beginning to emerge; when BDNF signaling is reduced, as occurs during activity blockade, there is an increase in the influence of excitatory neurons; when BDNF signaling is increased, as occurs during chronic increases in network activity, there is an increase in the influence of inhibitory neurons (Figure 2). When activity is blocked in cortical cultures using TTX, pyramidal cells become more excitable through increases in mEPSC amplitude [41], decreases in mIPSC or spontaneous IPSC amplitude [21, 49], and increases in the intrinsic cellular excitability of these cells [45]. All three of these compensatory changes appear to be mediated by reduced BDNF signaling because they are prevented by coapplication of BDNF and TTX and recapitulated by blocking BDNF signaling through its receptor, TrkB. On the other hand, increases in BDNF signaling that would be associated with overly active networks enhanced the influence of inhibitory interneurons through increases in interneuronal projections (increased mIPSC frequency), or through increased mEPSC amplitude onto inhibitory interneurons [20, 41]. While the model shown in Figure 2 is well supported by most of the experimental evidence, one exception to the homeostatic model is the observation that activity block triggers a BDNF-dependent increase in inhibitory interneuron intrinsic excitability in cortical cultures [45].

## 6. Sensors for Activity Perturbations

The sensors of activity that trigger homeostatic plasticity changes are a major focus in the field but are poorly understood. Activity sensors triggering changes in inhibitory circuitry are even less well understood. In the vast majority of homeostatic studies, network activity is reduced by TTX or glutamate receptor blockers or increased by GABA_A_ receptor antagonists. All of these treatments alter activity levels, as well as neurotransmission, throughout the network. Therefore, it is possible that changes in network spiking activity, cellular spiking activity, or synaptic transmission trigger homeostatic changes in mIPSCs. Very few studies exist that allow us to independently test these different triggers. One recent study increased spiking activity in an individual cell in an otherwise unperturbed network. The activity-increased cell exhibited increases in mIPSC amplitude and frequency [20]. The finding is consistent with the idea that increases in individual cellular spiking activity trigger homeostatic compensations of GABAergic inputs. However, when activity was blocked in individual hippocampal pyramidal cells by transfecting them with a K^+^ channel or a mutant voltage-gated Na^+^ channel, no change in mIPSC amplitude was observed [18]. The finding indicated that reductions in the activity of individual excitatory neurons did not trigger homeostatic changes in mIPSC amplitude, but suggested that reductions in network-wide activity or neurotransmission may be required to induce this plasticity. Consistent with the possibility that sensors assess neurotransmission, we have determined that *in vivo* blockade of depolarizing GABA_A_ transmission in the embryonic spinal network triggered compensatory increases in excitatory GABAergic mPSC amplitude and cellular excitability in motoneurons [50, 51]; these forms of compensatory plasticity were not dependent on alterations in spiking activity, suggesting that the network could sense reduced spiking activity levels through reduced GABA_A_ transmission, essentially using GABA as a proxy for activity levels. A better understanding of the sensors that trigger compensatory changes in inhibitory neurotransmission will require more extensive work than the current studies, but it will be important to consider the possibility that neurotransmission is involved in the process.

## 7. Concluding Remarks

Certain rules of our simplistic homeostatic plasticity model appear to be generally followed. GABAergic inputs to excitatory neurons in several different networks are strengthened following increases in activity and weakened following activity block; increases in activity lead to increased mEPSC amplitude in inhibitory neurons; increases in BDNF signaling (associated with increases in activity) increase the excitability of inhibitory interneurons, while decreases in BDNF signaling (associated with decreased activity) increase the excitability of excitatory neurons. However, there are several clear examples that do not fit into any simplistic homeostatic model (interneuron intrinsic excitability, mEPSC amplitude in interneurons following activity block, evoked responses between excitatory and inhibitory neurons). These apparent exceptions to the homeostatic model could arise for several reasons. It will be important to identify common mechanisms of homeostatic plasticity, but it is likely that different preparations (e.g., *in vitro* versus *in vivo*) and different neural circuits use different homeostatic mechanisms. Further, compensatory mechanisms will be experienced in different elements of the circuitry at different developmental stages [52]. In addition, the methods of altering network activity are likely to trigger different homeostatic mechanisms, for instance, increasing versus decreasing activity. In some cases, particularly *in vivo* studies, assumptions are made about alterations in cellular or network activity, but are not directly tested, leaving open the possibility that apparent antihomeostatic responses are actually homeostatic, or vice versa. It is also possible that in some cases absolute levels of spiking activity are not the homeostatic goal, but rather some more sophisticated pattern of activity, for instance, synchrony of the output neurons, which could be achieved through more complicated changes in GABAergic interneurons [53, 54]. In the end, it is important to recognize that changes in GABAergic synaptic strength or cellular excitability in inhibitory neurons are being tested in isolation, but they occur within complex networks where it is difficult to know the functional consequences of these changes. As the field matures it will be important to take these complexities into consideration. Because network-wide activity is clearly maintained across many neural circuits, there are likely to be strong homeostatic mechanisms maintaining global network activity; it will be important to differentiate these homeostatic mechanisms from those that maintain individual cellular activity or individual synaptic activity, each potentially being triggered by different sensors.

## Figures and Tables

**Figure 2:**